# Buffalo General Hospital

**Published:** 1858-08

**Authors:** 


*Buffalo General Hospital.*—A knowledge of the futile efforts which have been made to organize an institution of this kind, would give some idea of the work which has been accomplished, now that the "Buffalo General Hospital" is prepared to receive patients. These efforts are tradition to us, but the most prominent men in the profession have labored, and labored in vain until now, for the establishment of such an institution. In the early history of Buffalo, we believe about twenty years ago, land was donated for the purpose of establishing a hospital. This, however, was a failure. In 1847 the necessity of a hospital was too evident to admit of farther delay, and one was organized under a special charter. This met with the most violent opposition from some members of the profession, and an appropriation which was nearly obtained from the State, was lost. The next year, however, the Buffalo Hospital of the Sisters of Charity went into operation. This has gradually been enlarged until it has reached three times its original dimensions, and another addition is now being made. The Charity Hospital was of immense benefit to the city and to the profession. It is situated in the rear of the Buffalo Medical College, and affords ample opportunities for clinical instruction. These facilities, which have been taken advantage of by the faculty of the college, have placed that institution on a par with the colleges of the larger cities, and given it a position which it could never have attained without the advantages of bed-side instruction.

These facilities for the care of the sick, answered the purpose perfectly for a time; but the rapid growth of the city, the immense business which is yearly transacted on its wharves, canals, and railroads, made another hospital necessary. Appreciating this necessity, another attempt was made in 1854. Millard Fillmore was at the head of the head of the board of trustees, which consisted of fifty members. It was thought unadvisable to commence operations, however, without a capital of $100,000 to begin with, and as it was impossible to raise this amount, the project was abandoned.

This brief account will give some idea of the immense difficulty of starting an institution of this kind; but the friends of the hospital, in spite of the discouragement which they had suffered on all sides, and opposition where they could least have expected it, made the effort, the success of which we are proud as a citizen and a member of the profession of Buffalo, to chronicle. Justly believing that the board of trustees in the former instances had been unwieldly from the number of its members, they selected nine men, whose well-known activity and public spirit pointed them out as men who could carry the project through if they would, and being assured personally of their interest in the matter, appointed them trustees of the Buffalo General Hospital. On the twenty-first of November, 1855, the association was formed, a certificate of which was filed in the office of the Secretary of State, Dec. 12th, 1855, and in the clerk's office of Erie County, Dec. 13th, 1855. Subscriptions were immediately solicited, and in the winter of 1857, the hospital received an appropriation from the State of $10,000. The sum subscribed by our citizens amounted to $20,000.

The board of trustees have done a great work, and the magnificent building which they have commenced, will be a lasting monument to their industry and public spirit. There is yet another reward: the reflection that thousands of poor sufferers will be relieved in this institution, and will here find friends, perhaps in a strange land, will be a sufficient reward to those whose impulses prompted them to engage in this benevolent enterprise.

The building is situated on High street, with a front of 361 feet; a front of 450 feet on Goodrich street, and a depth of 282 feet. The situation is decidedly the finest in the city, having a most commanding view of the lake, the river and the surrounding country, and cooled in the warmest days of summer, by a delightful lake breeze. The west wing of the building has been completed, and is now ready for the reception of patients. It has four large and airy wards, which are much higher than usual, and private rooms, which are all ventilated by an ingenious and effective contrivance. Particular attention was properly paid to this important feature of the building. This wing is capable of accommodating upwards of one hundred patients, and the original design makes the part erected, one-third of the entire building which, when completed, will have a spacious amphitheatre, capable of holding two hundred persons, a room for a library and museum, and every convenience that could be desired. Arrangements have been made for the collection of a pathological museum, and for clinical instruction, and it is designed to extend charity, limited of course by the funds of the association, to all indigent persons requiring medical and surgical relief, irrespective of nation, race or religious creed. The nine men who have accomplished this great undertaking, are Charles E. Clarke, president of the board of trustees, Andrew J. Rich, vice-president, William Wardwell, secretary and treasurer, and George S. Hazard, Bronson A. Rumsey, Roswell L. Burrows, Stephen C. Howell, and Henry Martin. The trustees appointed the following gentlemen as the medical officers of the hospital for one year, dating from the 1st of July 1858:

*Attending Physicians*, Drs. Jas. M. Newman, Thos. F. Rochester, Cornelius C. Wyckoff.

*Consulting Physicians*, Drs. James P. White, Geo. N. Burwell, P. H. Strong.

*Attending Surgeons*, Drs. Chas. H. Wilcox, Sandford Eastman, Austin Flint, Jr.

*Consulting Surgeons*, Drs. Frank H. Hamilton, C. C. F. Gay, John Root.

The hospital was dedicated on the 26th of June, with interesting and appropriate ceremonies. A large number of prominent citizens were present on the occasion, demonstrating their great interest in the enterprise. The dedicatory poem was written for the occasion by one of our most talented and accomplished ladies, and was one of the most beautiful and appropriate efforts of the kind we have ever heard. The address was delivered by the Hon. James O. Putnam, and was listened to with marked attention. We quote the closing remarks, which contain a flattering tribute to the profession of this city:

"And in this connection it will not be deemed indelicate or invidious to say, that if this hospital, under your patronage, shall grow to its proposed dimensions, and as your city progresses in population, be filled with the diseased and disabled poor, it can never outgrow either the science or the self-sacrificing charity of the medical profession of Buffalo. I think I know something of the spirit which pervades that profession, and I feel that I can say, while they expect, and are entitled to, adequate compensation for services rendered those able to reward them, its heart, its hand, its science, are ever ready to answer the calls of the suffering poor.

"Citizens of Buffalo, the offering we this day dedicate, is yours to cherish and to place upon an enduring basis. It is one of the noblest that can be brought into the Temple of Humanity. That Temple is wide as the heavens, and receives within its portals every child of affliction and sorrow. That Charity which came to earth an angel-attendant upon the babe of Bethlehem, knows no distinction of caste, complexion, or nationality. She asks not at what altar the sufferer worships, and before she relieves, does not stop to inquire whether he even be a worshiper at all. And if she chance to find him without a faith and without a God, poor in soul as he is wretched in body, she delights, so far as comports with delicacy and propriety, in the double office of ministering to his temporal necessities, while with gentle guidance she points the wanderer 'to brighter worlds, and leads the way.' I seem to hear a voice coming up through the vale of the centuries, clear and resonant, 'GO AND DO THOU LIKEWISE.'"

---

*Obituary Notice.*—We are again called upon to notice the death of eminent professional men. Dr. Snow, who was so favorably known in connection with the use of anæsthetics, has lately died. We also learn of the death of Baron Boyer, the eminent surgeon of the Hotel Dieu of Paris.

